# CD151 promotes cell metastasis via activating TGF-β1/Smad signaling in renal cell carcinoma

**DOI:** 10.18632/oncotarget.24028

**Published:** 2018-01-08

**Authors:** Yajie Yu, Chao Liang, Shangqian Wang, Jundong Zhu, Chenkui Miao, Yibo Hua, Meiling Bao, Qiang Cao, Chao Qin, Pengfei Shao, Zengjun Wang

**Affiliations:** ^1^ Department of Urology, The First Affiliated Hospital of Nanjing Medical University, Nanjing 210029, China; ^2^ Department of Pathology, The First Affiliated Hospital of Nanjing Medical University, Nanjing 210029, China

**Keywords:** CD151, epithelial-to-mesenchymal transition, metastasis, renal cell carcinoma, TGF-β1/Smad

## Abstract

Tetraspanin CD151 has been identified as a tumor promoter, which is upregulated in various malignant cell types. However, the function of CD151 and its underlying mechanism in renal cell carcinoma is still unknown. In this study, we detected the expression of CD151 in RCC cells and tissues and explored its regulatory mechanism. We found that CD151 was upregulated in renal cell carcinoma tissues and cells and its expression was significantly associated with tumor stage (p=0.019) and survival (p=0.001) by analyzing tissue microarrays. After silencing of CD151 via lentivirus vector in Caki-1 and Caki-2 cells, reduced ability of migration and invasion were detected with downregulation of CD151. The opposite results were observed in cells with CD151 overexpression. Furthermore, western blotting was performed to investigate the influence of CD151 on epithelial-to-mesenchymal transition, matrix metalloproteinase 9 and TGF-β1/Smad signaling pathway in RCC. Subsequently, upregulating the protein level of transforming growth factor-β1 in cells with silencing of CD151 could rescue the malignant behaviors inhibited, which indicated that CD151 may play its promoting role in RCC partially by stimulating the expression of TGF-β1. Conclusively, CD151 might exhibit a prominent role in migration and invasion of RCC cells via activating TGF-β1/Smad signaling pathway.

## INTRODUCTION

Renal cell carcinoma (RCC) accounts for approximately 2-3% of all malignant diseases in adults [1]. Approximately 63,990 new cases were diagnosed with RCC and 14,400 deaths were estimated in the United States in 2017 [2]. And RCC is also a common malignant cancer in China, reported to have approximately 66,800 new diagnoses and 23,400 deaths in 2015 [3]. The causes of RCC are poorly understood, and the most effective method to treat RCC is nephrectomy because of its high resistance to chemotherapy and radiotherapy [4]. However, 20% of patients undergoing nephrectomy will have a relapse and develop metastatic RCC (mRCC) during follow-up [5]. Considering that targeted therapies for RCC (including bevacizumab, sorafenib, sunitinib and temsirolimus) have been applied to clinical treatment [6], the search for new diagnostic and therapeutic molecular markers for RCC has become important.

Tetraspanins are a family of membrane glycoproteins, with more than 30 members in mammals [7]. They are characterized by four transmembrane domains delimiting a small extracellular loop containing 20-30 amino acids and a large extracellular loop containing 70-150 amino acids. Tetraspanins are involved in modulation of multifarious normal physiological processes (e.g. cell activation, proliferation, adhesion and motility) and pathological processes (e.g. tumor progression and metastasis) [8]. As one of the most important members of the tetraspanin family, CD151 (also known as GP-27, MER 2, PETA-3, SFA-1 or Tspan24) was observed in different cell types [9]. High expression of CD151 gene and protein has been detected in tumour tissues from breast, colorectal, hepatocellular, lung, pancreatic and prostate [10–15] cancer patients. Altered expression of CD151 was involved in cancer growth, progression, invasion and metastasis [16–18]. There has been an increasing number of studies showing that high level of CD151 expression in tumor tissue was associated with cancer patients’ poor prognosis retrospectively [19]. In addition to being considered as a diagnostic and prognostic marker, its value as a therapeutic target has been shown in studies of CD151-specific antibodies [20]. However, there is few study with regard to CD151 expression and RCC. As a result, the relationship between CD151 and RCC remains unclear. Thus, analyzing the biological effects and underlying molecular mechanism of CD151 in RCC is significant.

CD151 has been considered as a positive regulator of the transforming growth factor β (TGF-β) signaling pathway [21]. TGF-β is a member of a superfamily with more than 40 secreted cytokines [22]. Plenty of studies have proved that TGF-β is associated with carcinogenesis and tumor progression. TGF-β has been reported to function as a tumor promoter by inducing epithelial-to-mesenchymal transition (EMT), which features the loss of epithelial phenotypes (such as claudins and E-cadherin) and the acquisition of mesenchymal phenotypes (such as fibronectin, N-cadherin and vimentin) [23]. With the assistance of EMT, epithelial cancer cells lose epithelial polarity and cell–cell adhesive junctions while reorganizing cytoskeleton and acquiring mesenchymal characteristics, which allow them to pass through the basement membrane and engender distant metastasis [23].

In this study, we used quantitative polymerase chain reaction (qPCR) and western blotting (WB) to examine the CD151 expression levels in RCC samples and RCC cell lines. Then, a series of *in vitro* assays have been conducted to figure out the relationship between CD151 and RCC. To further investigate how CD151 promotes cell migration and invasion by targeting TGF-β, we detected a number of hallmarks of EMT and TGF-β1/Smad signaling and performed a rescue experiment. Additionally, we applied tissue microarrays (TMAs) of RCC samples and immunohistochemistry (IHC) analyses to evaluate the correlation between the expression of CD151 and clinicopathologic characteristics of RCC patients.

The results of this study may reveal how CD151 acts as a tumor promoter in RCC cell lines and may provide a potential biomarker for the diagnosis, treatment and prognosis of RCC.

## RESULTS

### Upregulation of CD151 in RCC tissues and cell lines

**Figure 1:**
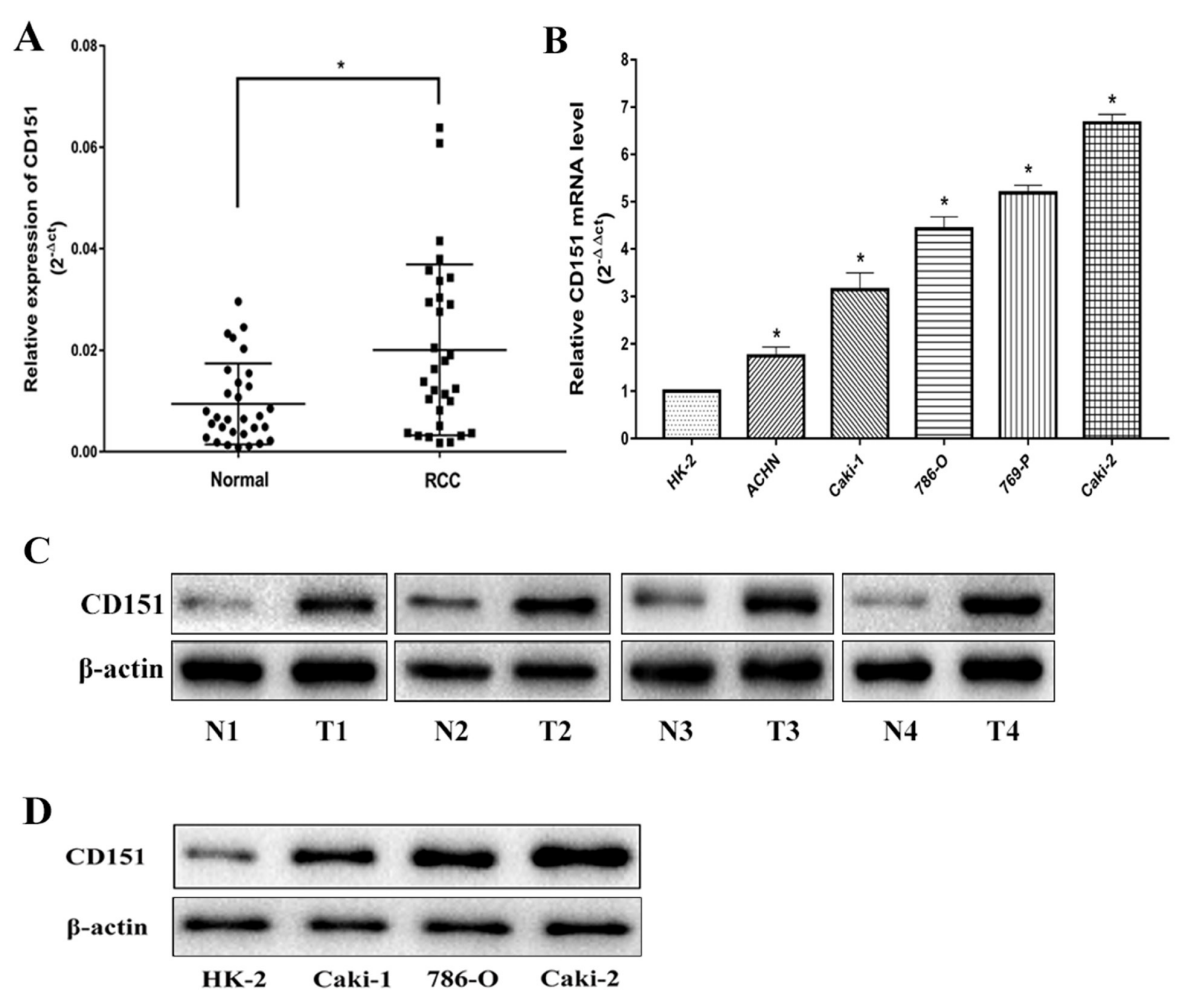
CD151 is upregulated in RCC tissues and cells **(A** and **C)** CD151 level in RCC samples was significantly upregulated compared with the paired adjacent normal tissues according to qRT-PCR and WB. **(B** and **D)** The expression level of CD151 in RCC cell lines was lower compared with the normal renal cell line according to qRT-PCR and WB. The median in each triplicate was used to calculate the CD151 expression using either the comparative 2^-ΔΔct^ or 2^-ΔCt^ method. ^*^ P < 0.05 compared with the adjacent normal tissues or HK-2 cell line.

Quantitative real-time polymerase chain reaction (qRT-PCR) was conducted to investigate the mRNA expression level of CD151 in 30 paired RCC tissues and adjacent normal tissues. In addition, we also detected the mRNA level of CD151 in five RCC cell lines (ACHN, Caki-1, 786-O, 769-P and Caki-2) and in the normal renal cell line (HK-2). The results showed that CD151 was significantly up-regulated in RCC tissues and five RCC cell lines, compared with that in adjacent normal tissues and HK-2 cell line, respectively (p < 0.05; Figure 1A, 1B).

Subsequently, the protein expression level of CD151 was examined by WB in tissues and cell lines. The results were consistent with that of qRT-PCR (p < 0.05; Figure 1C, 1D). All the results confirmed the upregulation of CD151 in RCC tissues and cell lines.

### Enhancement of CD151 on cell migration and invasion

**Figure 2:**
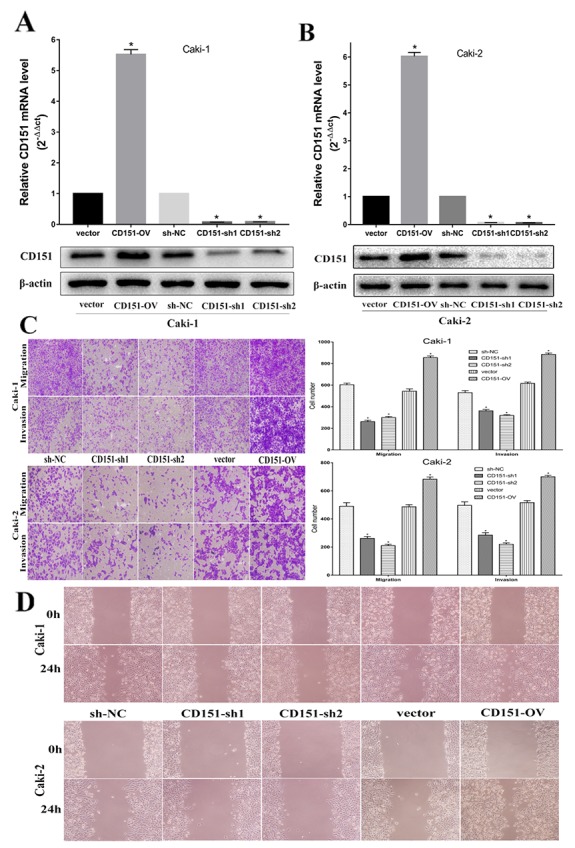
CD151 inhibits cell migration and invasion in the Caki-1 and Caki-2 cell lines **(A** and **B)** After transfection of lentiviral vector, CD151 expression was upregulated in CD151-OV group and downregulated in CD151-sh group in the Caki-1 and Caki-2 cell lines respectively. **(C** and **D)** Overexpression of CD151 promoted migration and invasion nevertheless knockdown of CD151 significantly inhibited the migration and invasion ability according to transwell assays and would healing assays. Data are mean ± SD of at least three independent experiments. ^*^ P < 0.05 compared with the negative control group. Original magnification ×200.

We constructed stable CD151 overexpressed (CD151-OV) and knocked-down (CD151-sh1/2) cell line by transfecting lentiviral vector and negative control (NC) group in Caki-1 and Caki-2, respectively. As shown in Figure 2, CD151 mRNA and protein expression were significantly upregulated in CD151-OV group and downregulated in CD151-sh1/2 group in Caki-1(p < 0.05; Figure 2A) and in Caki-2 (p < 0.05; Figure 2B) after transfection.

Migration, invasion and wound healing assays were performed to validate whether CD151 could affect migration and invasion ability in Caki-1 and Caki-2. Compared with negative control, overexpression of CD151 promoted the migration and invasion of Caki-1 cells. Conversely, knockdown of CD151 inhibited the ability of Caki-1 cells (p < 0.05; Figure 2C). The results of Caki-2 cells were consistent with that of Caki-1 cells (p < 0.05; Figure 2C).

These effects were confirmed by wound healing assays, which showed that overexpression of CD151 induced migration of Caki-1 and Caki-2 cells, whereas knockdown of CD151 inhibited migration of Caki-1 and Caki-2 cells, compared to negative control cells (p < 0.05; Figure 2D). These results suggested that CD151 may facilitate RCC cells metastasis.

### Regulation of CD151 on EMT, MMP-9 and TGF-β1/Smad signaling

**Figure 3:**
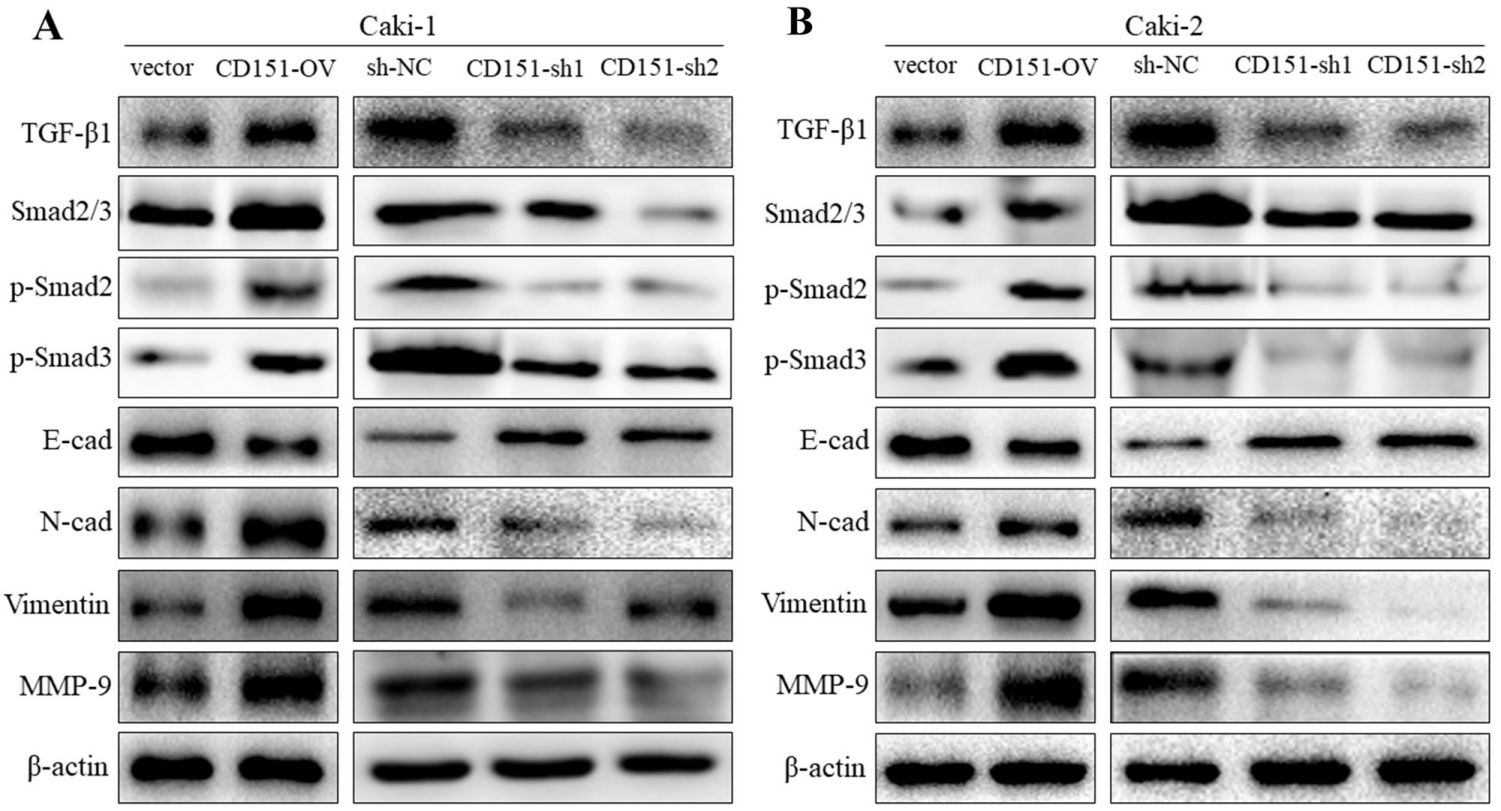
Regulation of CD151 on EMT, MMP-9 and TGF-β1 signaling Western blot analysis was used to detect the changes in MMP-9, TGF-β1 and EMT-related protein expression after transfection of lentiviral vector. **(A)** In Caki-1 cell line, E-cad was decreased while TGF-β1, p-Smad2, p-Smad3, N-cad, Vimentin and MMP-9 were increased in CD151-OV group cells. The results were opposite in CD151-sh1/2 group cells. **(B)** In Caki-2 cells, the similar results were detected. ^*^P < 0.05 compared with the negative control group.

To explore the molecular mechanism and pathway by which CD151 may influence cellular function in RCC cells, we detected the protein expression of matrix metalloproteinase-9 (MMP-9), TGF-β1 and EMT markers (including E-cadherin, N- cadherin and vimentin). In Caki-1 cell line, western blotting revealed a significant decrease and increase of E-cadherin with overexpression and downregulation of CD151 respectively, compared with NC group. The expression level of N-cadherin, vimentin, MMP-9 and TGF-β1 were higher in CD151-OV group than NC group. Inversely, compared with NC group, CD151-sh group had lower expression of N-cadherin, vimentin, MMP-9 and TGF-β1. Then, we detected the phosphorylation of Smad2 and Smad3(p-Smad2, p-Smad3), downstream of TGF-β1 by immunoblotting. Correspondingly, the changes were consistent with TGF-β1 (p < 0.05; Figure 3A). As for Caki-2 cell line, the outcome was in accordance with that of Caki-1 (p < 0.05; Figure 3B).

### Rescue experiment on cell migration and invasion

**Figure 4:**
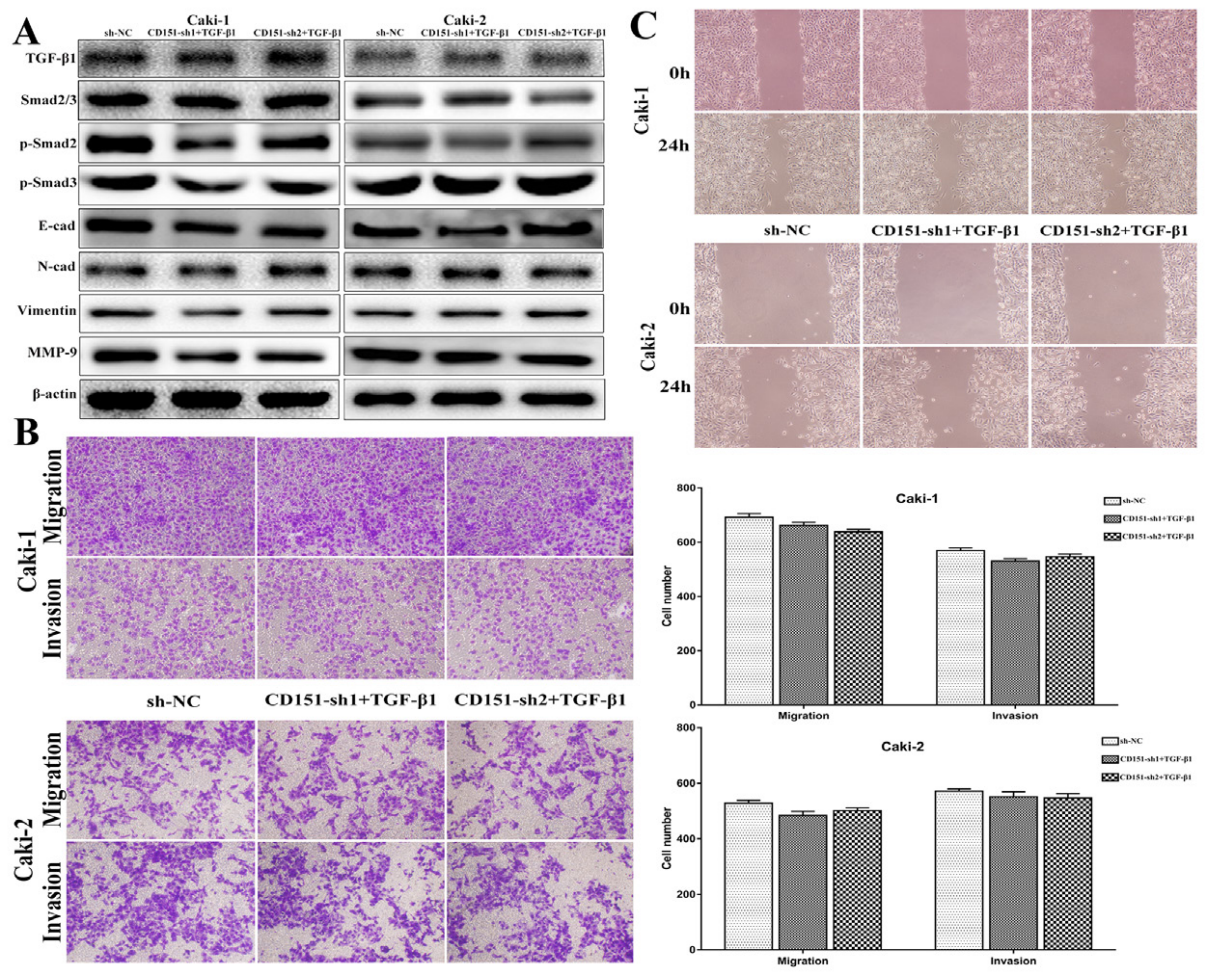
Observation on cell migration and invasion after Rh TGF-β1 stimulation in CD151-sh group cells **(A)** TGF-β1, p-Smad2, p-Smad3, E-cad, N-cad, Vimentin and MMP-9 protein expression in Caki-1 and Caki-2 cells were restored after Rh TGF-β1 stimulation. **(B** and **C)** The inhibitory role of CD151 downregulation on the migration and invasion of Caki-1 and Caki-2 cells was attenuated after Rh TGF-β1 stimulation. p > 0.05 compared with the negative control group.

After transfection of lentiviral vector and CD151 being knocked down, Caki-1 and Caki-2 cells were stimulated with recombinant human transforming growth factor-beta 1 (Rh TGF-β1) to elevate the protein level of TGF-β1 to testify if RCC cellular behavior could be rescued by restoring TGF-β1 expression. After Rh TGF-β1 stimulating, we isolated protein from cells and detected the expression of TGF-β1, p-Smad2, p-Smad3, E-cadherin, N-cadherin, vimentin and MMP-9 by immunoblotting. As shown in Figure 4A (p > 0.05), TGF-β1 expression level was raised in Caki-1 and Caki-2 cells and the levels of p-Smad2, p-Smad3, N-cadherin, vimentin and MMP-9 were restored subsequently. Correspondingly, E-cadherin protein expression was suppressed.

Using the transwell assays, decreased migration and invasion capability of Caki-1 and Caki-2 cells induced by CD151 downregulation was reversed after Rh TGF-β1 motivating. The results showed no significant difference between NC group and sh+TGF-β 1 group (p > 0.05, Figure 4B).

Furthermore, the results of wound healing assays were consistent with those of the transwell assays (p > 0.05, Figure 4C). The restricted migration ability of CD151-downregulated Caki-1 cells was rescued, compared with NC group. Accordingly, the same effect was found in Caki-2 cell line.

These results indicated that the promotive role of CD151 in RCC might function partially through adjustment of TGF-β1/Smad signaling.

### Relationship between CD151 expression and clinicopathologic characteristics of RCC patients

**Figure 5:**
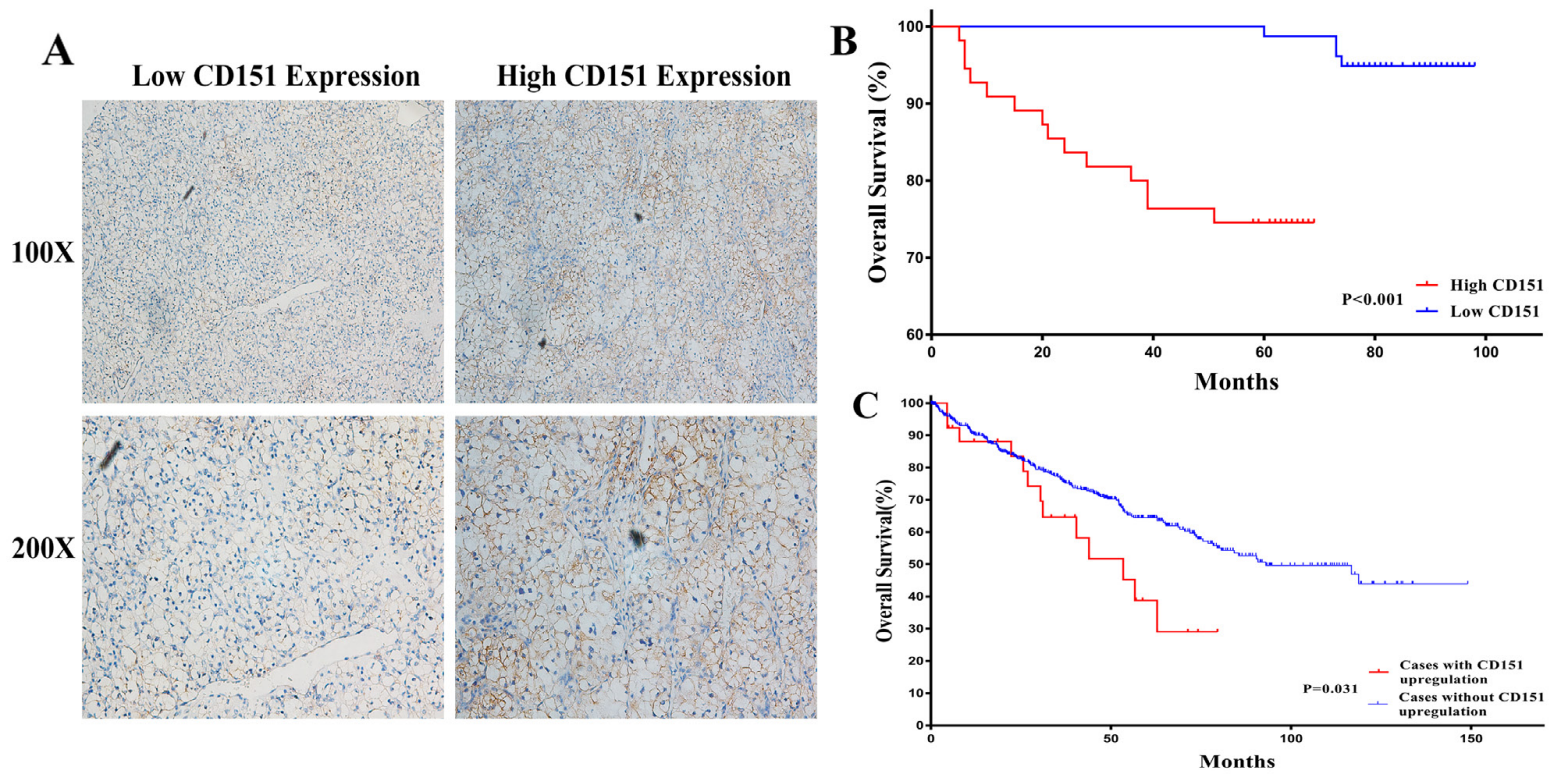
Human RCC TMAs were used to assess the relationship between CD151 and clinicopathologic characteristics of RCC patients **(A)** CD151 was mainly expressed in the cytomembrane of RCC cells and low/high CD151 expression of immunohistochemical staining were shown. **(B)** High CD151 expression showed significance with lower overall survival in RCC patients by univariate Kaplan-Meier/log-rank analysis in our study. P < 0.001 **(C)** Correlation between status of CD151 gene and overall survival from TCGA RCC samples. P = 0.031

The protein expression of CD151 of 133 clinical RCC tissues was detected via TMAs and IHC technique to further investigate the association between CD151 and RCC. The IHC results showed that CD151 proteins were mostly expressed in the cytomembrane of RCC cells (Figure 5A). Characteristics of the 133 RCC patients involved in this study were showed in Table 1 and the high expression rate of CD151 was 41.4% (55 of the 133 RCC tissues). To evaluate the clinical significance of aberrant CD151expression, the patient cohort was divided into CD151-low and CD151-high groups, which was based on the proportion and intensity of immunostaining. As shown in Table 2, we found that advanced tumor stage and poor survival were significantly associated with high CD151 level (p < 0.05). Nevertheless, no significant differences were revealed between age, gender, tumor size, histological grade and expression of CD151.

**Table 1:** Characteristics of the 133 RCC patients involved in this study

Age		
Mean±SD, year	56.34±13.73	
<60	48	36.1%
≥60	85	63.9%
Gender		
Male	83	62.4%
Female	50	37.6%
Tumor size		
Mean±SD, cm	4.81±2.56	
≤4	71	53.4%
>4	62	46.6%
Histology		
Clear cell carcinoma	118	88.7%
Others	15	11.3%
Histological grade		
I	28	21.0%
II	82	61.7%
III	19	14.3%
IV	4	3.0%
Tumor stage		
T1	115	86.5%
T2	11	8.3%
T3	6	4.5%
T4	1	0.8%
Survival		
Mean±SD, month	70.40±19.97	
No	18	13.5%
Yes	115	86.5%
CD151 expression		
Low	78	58.6%
High	55	41.4%

SD, standard deviation.

**Table 2:** Relationship between CD151 expression and clinicopathologic characteristics of patients

Variable	Total (%)	CD151 expression		
		Low (n=78)	High (n=55)	*P* value
Age (year)				0.498
<60	48	30 (62.5)	18 (37.5)	
≥60	85	48 (56.5)	37 (43.5)	
Gender				0.906
Male	83	49 (59.0)	34 (41.0)	
Female	50	29 (58.0)	21 (42.0)	
Tumor size (cm)				0.563
≤4	71	40 (56.3)	31 (43.7)	
>4	62	38 (61.3)	24 (38.7)	
Histology				0.657
Clear cell carcinoma	118	70 (59.3)	48 (38.1)	
Others	15	8 (33.3)	7 (66.7)	
Histological grade				0.820
I-II	110	65 (59.1)	45 (40.9)	
III-IV	23	13 (56.5)	10 (43.5)	
Tumor stage				**0.019**
T1	115	72 (62.6)	43 (37.4)	
T2-T4	18	6 (33.3)	12 (66.7)	
Survival				**0.001**
No	18	4 (22.2)	14 (77.8)	
Yes	115	74 (64.3)	41 (35.7)	

Bold: *P* < 0.05. *P* values were two-tailed and based on the Pearson chi-square test.

According to Univariate Kaplan-Meier/log-rank analysis, there was significant difference between high CD151 protein expression and poorer clinical prognosis in RCC patients (log-rank p < 0.001; Figure 5B). A similar trend was also detected between CD151 gene status and poor overall survival in The Cancer Genome Atlas (TCGA) RCC dataset (log-rank p=0.031; Figure 5C).

## DISCUSSION

In view of that tetraspanins are likely to function as a part of larger molecular aggregates involved in the control of cell proliferation and migration [24], efforts are now being directed towards the identification of specific functional partners for individual tetraspanin. CD151, as one of the most important members of the tetraspanin family, has been found high expressed in numerous neoplasms [10–15]. A growing body of evidence have showed that changed expression of CD151 is related with cancer progression, invasion and metastasis [17, 18]. Wang et al reported that CD151 induced osteosarcoma metastasis likely by regulating cell function through adhesion signaling [25]. CD151 can also interact with other tetraspanin family members (e.g. CD9, CD81) and integrins to regulate migration, adhesion and signaling of cells [26, 27].

Although a number deal of studies have shown that CD151 functions as a promoter gene in various carcinomas, the function of CD151 in RCC and the underlying mechanism has not been studied. Only few studies have reported that CD151 might characterize metastatic potential in Clear Cell Renal Cell Carcinoma (ccRCC) and could be a prognostic marker for progression of ccRCC [28, 29]. Our study demonstrated that CD151 could be an important indicator of RCC progression, and it might be involved in RCC metastasis.

We confirmed the upregulation of CD151 in RCC tissues and cell lines by qRT-PCR and WB, and the immunohistochemistry results of TMAs showed that CD151 proteins were mainly expressed in the cytomembrane of RCC cells. Furthermore, high CD151 expression was found to be significantly associated with advanced tumor stage and poor survival. This may provide evidence that the upregulation of CD151 could lead to RCC progression and metastasis. In the present study, to investigate the potential biological effect of CD151 on RCC cytological behaviors, we changed CD151 expression by lentiviral vector in RCC cell lines. It was found that high expression level of CD151 could promote migration and invasion ability of RCC cells via transwell assays and wound healing assays. Consequently, we suggest that the aberrant expression of CD151 could act as a tumor promoter and therapeutic target for treating RCC.

Metastasis of cancer cells involves various cytophysiological changes (e.g. EMT) and the degradation of the extracellular matrix (ECM) by increasing and activating extracellular proteases, such as MMPs, urokinase-type plasminogen activator (u-PA), or serine proteinase, which has been regarded as important in cancer invasion and metastasis processes [30]. As reported, MMPs are the most essential proteases in the proteolysis of ECM proteins (e.g. collagen, elastin, fibronectin, laminin and proteoglycan) [31, 32]. Among MMPs family, MMP-9 is an important member. There is a growing body of evidence supporting that TGF-β1 plays a significant role in inducing MMPs through various signaling pathways [33, 34]. TGF-β1 has been recognized to function as a tumor promoter by inducing EMT, which features the loss of epithelial phenotypes (e.g. E-cadherin) and the acquisition of mesenchymal phenotypes (e.g. N-cadherin and Vimentin) [23]. Therefore, we conjectured that CD151 could affect the expression of TGF-β1 and then attenuate the TGF-β1-induced MMPs expression, thus activate tumor metastasis. To verify the assumption, we detected the expression of TGF-β1 and its signaling pathway-related biomarkers, and E-cad, N-cad, Vimentin and MMP-9 after transfection of lentiviral vector. When CD151 was silenced or overexpressed artificially in our study, the changes of p-Smad2/3 expressions were in line with TGF-β1, which suggested that CD151 might play a regulation role of TGF-β1/Smad signaling in RCC cell line. Our data showed that upregulation of CD151 notably enhanced the protein expression levels of TGF-β1. Accordingly, the expressions of N-cad, Vimentin and MMP-9 were raised, and that of E-cad was depressed. The opposite results were found in CD151-knockdown cell lines. Further, to confirm whether CD151 attributed its tumor promoting role to TGF-β1 in RCC or not, we stimulated the CD151-sh group cells with Rh TGF-β1 to restore the TGF-β1 expression. TGF-β1/Smad signaling could be revived and induced inhibitory effect on cell migration and invasion was impaired by Rh TGF-β1, indicating that downregulation of CD151 inhibited the activation of TGF-β1/Smad signaling pathway instead of completely suppressing it.

The conclusion of this study is incongruous, even incompatible with the paper published by Johnson JL et al [35]. In that paper, the authors found that CD151 could regulate RhoA activation and the dynamic stability of carcinoma cell-cell contacts. Enhanced migration of CD151-silenced carcinoma cell sheets was observed in their experiments. However, the authors failed to determine whether the ability of CD151 to promote integrin-dependent cell motility and its ability to promote stable cell-cell junctions are separable functions. If so, factors regulating the balance between these opposing functions might determine whether CD151-integrin complexes act as promoters or suppressors of metastasis in specific settings. Collectively, the function of CD151 is dependent on the specific microenvironment of different carcinoma.

Although we have demonstrated the regulatory role of CD151 in TGF-β1/Smad signaling pathway which is first detected in RCC cell line. However, our study still has several limitations. The specific mechanism underlying this posttranscriptional regulation still needs to be investigated by further studies. What’s more, *in vivo* assays are required to confirm the promoting role of CD151 in RCC in the future.

In summary, our results indicated that CD151 is upregulated in RCC and high level of CD151 is associated with advanced tumor stage and poor survival significantly. In addition, we proved CD151 as a potential tumor promoter in RCC by activating TGF-β1/Smad signaling and EMT, and playing a crucial role in RCC cells migration and invasion. Consequently, CD151 might serve as a potential biomarker for the diagnosis, treatment and prognosis of RCC.

## MATERIALS AND METHODS

### Patients and tissue microarrays

Following the Local Ethics Committees of the First Affiliated Hospital of Nanjing Medical University, 30 paired tumor specimens and adjacent normal tissue samples used to assess CD151 expression were obtained with informed consent from RCC patients. All the patients had undergone partial nephrectomy or radical nephrectomy. All samples were obtained during surgery, immediately frozen in liquid nitrogen, and stored at −80°C for further analysis. The identification of tumor tissues and adjacent normal tissues were confirmed by the pathologists.

Two human RCC TMAs containing 133 RCC tissues were obtained from patients who were treated by partial or radical nephrectomy between 2008 and 2011 at the First Affiliated Hospital of Nanjing Medical University (Nanjing, China). All patients were recruited following informed consent and the protocols used in the study was approved by the medical ethics committee of the hospital. The TMA’s accompanying pathological data are described in Table 1. The follow-up deadline was April 2016. From each RCC tissue, triplicate tissue cores with diameters of 0.6mm were represented.

### Immunohistochemistry

Serial sections from TMA blocks were deparaffinized in xylene and rehydrated through an ethanol gradient, then were blocked in hydrogen peroxide in methanol for 10 min. Antigen retrieval was performed by incubation for 2 min in a steam pressure cooker containing citrate buffer 10 mM, pH 6.0. Then samples were blocked for 5 min and incubated overnight with primary antibodies against CD151 (Sigma, USA) (1:100) at 4°C overnight. After having been washed by phosphate buffer saline (PBS) for 10 min, slides were cultured in the secondary antibody for 30 min. After a 10 min wash in PBS, the antibody reaction was visualized with a fresh substrate solution containing DAB. The sections were counterstained with hematoxylin, dehydrated, and coverslipped.

### Evaluation of staining

The immunohistochemical staining was evaluated by two experienced pathologists without knowledge of the clinical data separately. The percentage of positive tumor cells was determined in at least five areas at ×200 magnification and assigned to one of the following five categories: 0, <5%; 1, 5–20%; 2, 20–50% and 3, 50–100%. Meanwhile, the intensity of immunostaining was scored as follows: 0, undetected; 1, yellow and 2, brown. By multiplying the two scores, the final immunohistochemical scores of RCC tissues for CD151 were: low expression (≤1) and high expression (2-5).

### Cell culture and Cell transfection

The human RCC cell line (ACHN, Caki-1, 786-O and 769-P) and normal human proximal tubular cell line (HK-2) were purchased from the Cell Bank Type Culture Collection of the Chinese Academy of Sciences (Shanghai, China), and human RCC cell line(Caki-2) was purchased from the China Infrastructure of Cell Line Resources. Cells of the Caki-1 and Caki-2 were maintained in McCoy’s 5A (Gibco, USA); cells of 786-O and 769-P were maintained in RPMI 1640 medium; cells of HK-2 were maintained in Dulbecco’s modified Eagle’s medium (Gibco, USA), all supplemented with 10% fetal bovine serum (FBS, Gibco, USA) within a humidified atmosphere containing 5% CO2 at 37°C.

The lentiviral vector with knock-down and overexpression of CD151 and a lentiviral vector alone used as a negative control were constructed by Genechem (Shanghai, China). Cells with CD151 knock-down were defined as CD151-sh group and cells overexpressing CD151 were defined as CD151-OV group, while cells transfected with lentiviral vector alone were defined as NC group. Cells of the Caki-1 and Caki-2 were seeded in 6-well plates at 40% confluence on the day before transfection. For viral infection, titrated viral stocks were suitably diluted in complete medium to obtain the desired multiplicity of infection (MOI) and added to Caki-1 and Caki-2 cell monolayers. Green fluorescent protein (GFP) expression could be detected to assess the infection efficiency 3 days after infection. Five days after infection, cells were harvested into two parts. RT-PCR was performed to evaluate CD151 expression efficiency in one part of cells, while the other part was kept for cell amplification and follow-up experiments.

### RNA extraction, reverse transcription polymerase chain reaction (RT- PCR) and quantitative real-time polymerase chain reaction (qRT-PCR)

The RNA was extracted from cell lines and tumor samples using Trizol reagent (Invitrogen, USA) and spectroscopy was used to detect the purity and concentration of the RNA. Total RNA was reversed transcribed into cDNA using PrimeScript RT Master Mix (Takara, JPN). Analysis of CD151 expression was performed by qRT-PCR using a SYBR Green assay according to the manufacturer’s instructions (Applied Biosystems, USA). We used the following primers (Realgene, Nanjing, China): CD151 forward, 5’-CTCACAGGACTGGCGAGAC-3’ and reverse, 5’-ACAGCCCCAATGACCCTCA-3’; β-actin forward, 5’-TGACGTGGACATCCGCAAAG-3’ and reverse, 5’-CTGGAAGGTGGACAGCGAGG -3’. Relative expression of CD151 were calculated using the 2^−ΔΔct^ method. Each reaction was run in triplicate.

### Protein isolation and western blot

Cells and human RCC tissues were lysed in radioimmunoprecipitation assay (RIPA) buffer (Beyotime, Beijing, China), supplemented with protease inhibitors (Roche, Shanghai, China) and the serine protease inhibitor phenylmethylsulfonyl fluoride (PMSF; Roche), at 4°C for 30min. The cell supernatants were extracted after centrifugation for 15 min at 14,000 rpm, then the concentration of the protein was determined using a BCA Protein Quantification kit (Beyotime Institute of Biotechnology). Proteins from tissues and cells were separated using 10% SDS-PAGE, transferred onto PVDF membranes (Millipore, Billerica, USA), blocked for 2h with 5% nonfat milk at room temperature, and incubated with primary antibodies at 4°C overnight. After that, the membranes were washed three times with TBST and incubated with a horseradish peroxidase-conjugated secondary antibody for 2 h at room temperature. Blots were detected using a Bio-Rad Bioimaging system (Bio-Rad, CA, USA), and antibodies against β-actin served as a negative control. Rabbit monoclonal antibodies (1:1000) against TGF-β1, E-cad, N-cad, Vimentin and MMP-9 (Cell Signaling Technology, USA) were used in Western blot analysis according to the manufacturer’s instructions.

### Transwell migration and invasion assays

For the migration assays, 2×10^4^ cells in 200μl of serum-free medium were placed in the top chamber of the transwell (pore size, 8 mm; BD Bioscience). For the invasion assays, 5×10^4^ cells in 200μl of serum-free medium were placed in the top chamber adhered with Matrigel (BD Bioscience) following the manufacturer’s protocol. While the lower chamber was filled with 20% FBS-containing medium. After incubation at 37°C for 24 hours, the cells on the upper surface were erased by a cotton swab, while the cells invaded on the bottom of membranes were fixed in 4% methanol solution and stained with 0.5% crystal violet and counted under a microscope. Five fields were randomly counted. Experiments were repeated 3 times.

### Recombinant Human TGF-β1 stimulation

Recombinant Human TGF-β1 (Rh TGF-β1) (R&D system, USA) was reconstituted at 20 μg/mL in sterile 4 mM HCl containing 0.1% bovine serum albumin for storing and use. The stimulating concentration to the CD151-sh group cells was 2ng/ml when cells were seeded in 6-well plates at 50% confluence. Cells were harvested for further transwell assays and protein isolation 48h after the stimulation. CD151-sh group cells stimulated with Rh TGF-β1 were defined as CD151-sh +TGF-β1 group.

### Statistical analyses

The relationship between CD151 protein expression and clinicopathological factors was evaluated by χ2-test. We used Kaplan–Meier curve and log-rank tests to assess the association between CD151 expression and overall survival. All data values were presented as mean ± SD and statistical analyses were performed using the SPSS20.0 software. P < 0.05 was considered statistically significant.
